# Selection of Thai Medicinal Plants with Anti-Obesogenic Potential via In Vitro Methods

**DOI:** 10.3390/ph13040056

**Published:** 2020-03-29

**Authors:** Wijitrapha Ruangaram, Eisuke Kato

**Affiliations:** 1 Division of Applied Bioscience, Graduate School of Agriculture, Hokkaido University, Kita-ku, Sapporo, Hokkaido 060-8589, Japan; wijit@chem.agr.hokudai.ac.jp; 2 Division of Fundamental AgriScience and Research, Research Faculty of Agriculture, Hokkaido University, Kita-ku, Sapporo, Hokkaido 060-8589, Japan

**Keywords:** medicinal plants, obesity, in vitro, lipase, 3T3-L1 cell, lipolysis, lipogenesis

## Abstract

The prevalence of obesity is increasing globally. Despite the availability of a variety of anti-obesogenic drugs, including therapies under clinical development, these treatments are often indicated for patients with severe obesity, making them unsuitable for patients with mild obesity or for preventative use. In Thailand, traditional remedies employing medicinal plants are widely used to maintain health and treat disease. These treatments are generally inexpensive and readily available at markets, making them good treatment options for preventing obesity. To evaluate the anti-obesogenic potential of Thai medicinal plants, we employed three in vitro methods: pancreatic lipase inhibition, lipolysis enhancement, and lipid accumulation reduction assays. Among 70 Thai medicinal plants, *Eurycoma longifolia* Jack, *Tiliacora triandra* Diels, and *Acacia concinna* (Willd.) DC. were selected as the most favorable candidates because they exhibited anti-obesogenic activity in all three assays. These medicinal plants are expected to have efficient anti-obesogenic effects, making them promising candidates for further study.

## 1. Introduction

According to the World Health Organization, the prevalence of obesity has been increasing globally [1]. In 2017, 2 billion and 650 million people were estimated to be overweight or obese, respectively. Thailand is an example of a country with a growing obese population [2]. Thailand has the second largest prevalence of obesity among Southeast Asian countries, and approximately 32% of the population is overweight [3]. Several drugs have been developed to treat obesity in conjunction with diet control and exercise [4]. Drugs exert positive effects on weight by inhibiting the absorption of lipids from the gastrointestinal tract or reducing food intake through suppressing appetite. However, some of the currently available anti-obesogenic drugs have been withdrawn from the market due to their adverse effects [4]. Hence, new drugs are continuously being studied.

Anti-obesogenic drugs are mainly prescribed for patients with severe obesity. Thus, alternative methods are needed for people with mild obesity or for those who want to prevent obesity. Traditional remedies that utilize various medicinal plants represent one treatment option for such people. Whereas regular exercise or diet modification to avoid obesity can be difficult for individuals, the consumption of medicinal plants is relatively easy, making them good alternatives. Additionally, various types of plants are used as traditional remedies in Thailand, and because several medicinal plants are available in Thailand, they are inexpensive compared with developed medicines. In addition, medicinal plants are generally used as food ingredients in Thailand, making them familiar and readily available from markets [5].

Currently, there is limited knowledge concerning the anti-obesogenic potential of medicinal plants in Thailand. Thus, this study aimed to evaluate the anti-obesogenic potential of Thai medicinal plants which are readily available in the markets in Thailand (Table 1). Most research for this purpose uses a single bio-assay to evaluate the potential of each plant to select a suitable candidate for further research. However single bio-assays may only reflect a part of the therapeutic potential. In the human body, multiple and complex steps are related to the development of obesity. Thus, by combining multiple bio-assays, the results are more likely to reflect the potential of medicinal plants and they will provide more valid information, which will increase the chance of obtaining successful results in further studies such as in vivo and clinical experiments. Thus, in this study, we used three in vitro assays (pancreatic lipase inhibition assay, lipolysis enhancement activity assay, and lipid accumulation reduction assay) to evaluate the anti-obesogenic potential of Thai medicinal plants and select the best candidates.

## 2. Results

### 2.1. Screening

Seventy Thai medicinal plant extracts were screened for their ability to inhibit pancreatic lipase activity at a concentration of 0.5 mg/mL, enhance lipolysis, and reduce lipid accumulation at a concentration of 0.05 mg/mL in order to select initial candidates. Each method had a specific criterion for efficacy as written below. Plants exhibiting activity in at least two in vitro assays were considered candidates for further analysis. The detailed results of these assays are summarized in Table S1.

#### 2.1.1. Pancreatic Lipase Inhibition

Pancreatic lipase is involved in the digestion of lipids in food, which represent one of the major causes of obesity. Inhibition of this enzyme decreases the absorption of lipids, and this strategy is known to effectively prevent obesity. The inhibitory activities of 70 Thai plant extracts against porcine pancreatic lipase were examined. The criterion for positivity in this assay was an inhibition rate higher than 55%, which is the average inhibition rate of cetilistat, the positive control used in this experiment. Twenty-seven extracts met this criterion: *Tinospora crispa* (L.) Hook. f. and Thomson, *Thunbergia laurifolia* Lindl., *Momordica charantia* L., *Amomum testaceum* Ridl., *Lagerstroemia speciose* (L.) Pers., *Andrographis paniculate* (Burm.f.) Nees, *Pluchea indica* (L.) Less., *Barleria lupulina* Lindl., *Syzygium aromaticum* (L.) Merr. and L.M.Perry, *Piper retrofractum* Vahl, *Curcuma aeruginosa* Roxb., *Morus alba* L., *Cymbopogon nardus* (L.) Rendle, *Ventilago denticulate* Willd., *Zingiber montanum* (J.Koenig) Link ex A. Dietr, *Boesenbergia rotunda* (L.) Mansf., *Erythrina subumbrans* (Hassk.) Merr., *Glycyrrhiza glabra* (L.), *Peltophorum pterocarpum* (DC.) K. Heyne, *Bridelia ovata* Decne., *Cleome viscosa* L., *Cladogynos orientalis* Zipp. Ex Span., *Dracaena cochinchinensis* (Lour.) S.C.Chen, *Wrightia arborea* (Dennst.) Mabb., *Cyperus rotundus* L., *Acacia concinna* (Willd.) DC., and *Caesalpinia sappan* L.

#### 2.1.2. Lipolysis Enhancement

Lipids are stored in adipocytes, and their levels in adipocytes are directly correlated with the obesity status of a person. The amount of lipids stored in adipocytes is controlled by the balance between lipogenesis and lipolysis. Thus, inhibiting lipogenesis or enhancing lipolysis will decrease the amount of lipids in adipocytes and exert favorable effects for treating or preventing obesity. 3T3-L1 adipocytes were used to evaluate the lipolysis-enhancing activities of Thai medicinal plant extracts, and the criterion for positivity was a greater than 30% increase in lipolysis compared with the effects of the control. Nine extracts satisfied this criterion: *Terminalia chebula* Retz., *Rauvolfia serpentine* (L.) Benth. Ex Kurz, *Cissus quadrangularis* L., *Chromolaena odorata* (L.) R.M.King and H.Rob., *Tiliacora triandra* Diels, *C. nardus*, *Eurycoma longifolia* Jack, *C. rotundus*, and *A. concinna*.

#### 2.1.3. Lipid Accumulation Suppression

The accumulation of lipids in 3T3-L1 adipocytes was measured to evaluate the effect of Thai medicinal plant extracts on lipogenesis. Although lipolysis enhancement also reduces lipid levels in adipocytes, assessment of both lipolysis enhancement and lipid accumulation suppression will clarify whether inhibition of lipogenesis or enhancement of lipolysis is the cause of reduced lipid accumulation in 3T3-L1 adipocytes. The criterion for positivity was a greater than 10% decrease in lipid accumulation compared with the control findings, and seven extracts satisfied this criterion: *M. alba*, *Ocimum tenuiflorum* L., *T. triandra*, *E. longifolia*, *C. rotundus*, *Jasminum sambac* (L.) Aiton, and *C. sappan*.

### 2.2. Anti-Obesogenic Potential of Identified Candidate Plants

The results identified seven medicinal plants with satisfactory effects in at least two of the three assays. The selected medicinal plants were *M. alba*, *T. triandra*, *C. nardus*, *E. longifolia*, *C. rotundus*, *A. concinna*, and *C. sappan*. These plants were subjected to further analyses using the same methods with different concentrations to more precisely reveal their potential. A cell viability assay was also performed to detect the cytotoxic effects of the extracts.

#### 2.2.1. Pancreatic Lipase Inhibition by the Candidate Extracts

**Figure 1:**
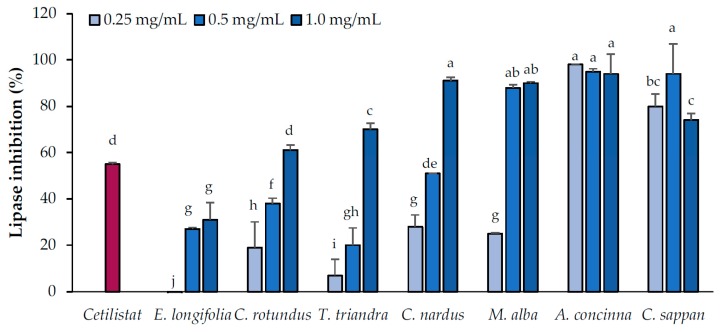
Pancreatic lipase-inhibitory activities of the candidate plant extracts. Cetilistat (positive control) concentration was 8 µM. Data are expressed as means ± standard deviation. Means with different letters are significantly different (Tukey’s test, *p* < 0.05).

The inhibitory effects of the candidate plant extracts on pancreatic lipase activity were evaluated at concentrations of 0.25, 0.5, and 1.0 mg/mL (Figure 1). Based on the results, the candidate plants were divided into three groups according to their pancreatic lipase-inhibitory activity (low, moderate, and high). The group with low inhibitory activity, consisting of plants that inhibited lipase activity by no more than 70% at the highest tested concentration, consisted of *E. longifolia* (31% ± 7.4%), *C. rotundus* (61% ± 2.3%), and *T. triandra* (70% ± 2.7%). The plants with moderate inhibitory activity, which inhibited lipase activity by more than 70% at the highest concentration but not at lower concentration, included *M. alba* (90% ± 20.6% at 1.0 mg/mL) and *C. nardus* (91% ± 1.5% at 1.0 mg/mL). The group with high inhibitory activity, which inhibited lipase activity by more than 70% at all concentrations, consisted of *A. concinna* and *C. sappan*.

#### 2.2.2. Lipolysis Enhancement by the Candidate Extracts

**Figure 2:**
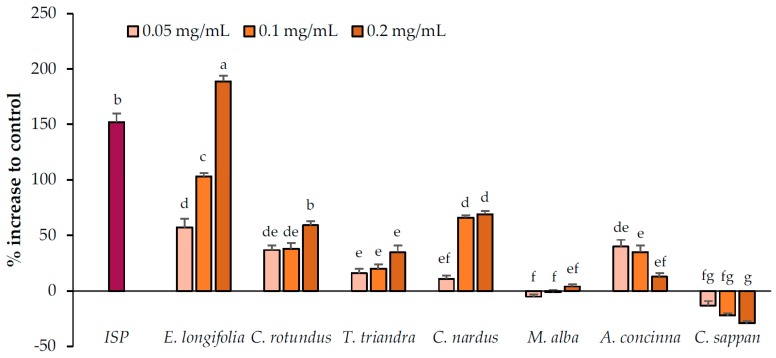
Lipolysis enhancement activity of the candidate plant extracts. Isoproterenol (ISP, positive control) was used at 2.5 µM. Data are expressed as means ± standard deviation. Means with different letters are significantly different (Tukey’s test, *p* < 0.05).

**Figure 3:**
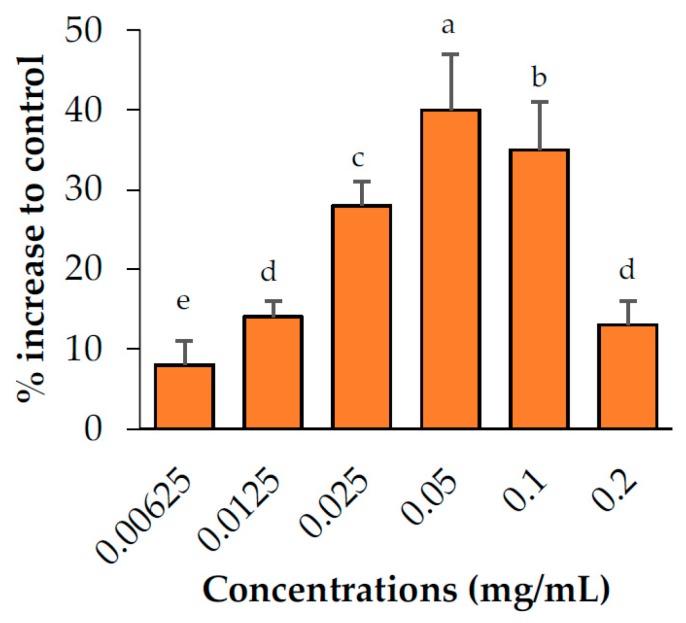
Lipolysis enhancement activity of the *Acacia concinna* extract. Data are expressed as means ± standard deviation. Means with different letters are significantly different (Tukey’s test, *p* < 0.05).

The abilities of the candidate plant extracts to enhance lipolysis were analyzed at concentrations of 0.05, 0.1, and 0.2 mg/mL (Figure 2). Based on the results, the extracts were categorized into three groups (no, relatively weak, and relatively strong activity). Two extracts, namely *M. alba* and *C. sappan* exhibited no ability to enhance lipolysis, whereas *T. triandra* and *A. concinna* exhibited relatively weak activity. Of these plants, a negative correlation was observed between the concentration of *A. concinna* and its lipolysis-enhancing effects. Cell viability testing (Figure 5) suggested that cytotoxicity was the cause of this negative correlation. Thus, lower concentrations (0.00625, 0.0125, and 0.025 mg/mL) were used for *A. concinna*, and the results revealed a positive correlation at these concentrations, confirming the interference of cytotoxicity at high concentrations (Figure 3). Three plants namely, *C. nardus*, *C. rotundus*, and *E. longifolia*, increased lipolysis by more than 50% relative to the control. We would like to note that *E. longifolia* displayed the highest activity among the seven candidate plants.

#### 2.2.3. Lipid Accumulation Suppression by the Candidate Extracts

**Figure 4:**
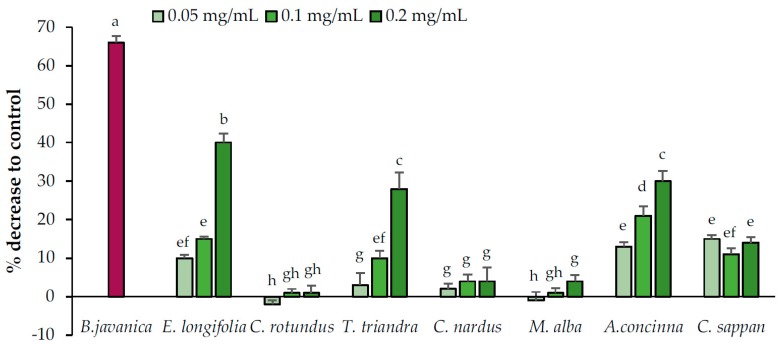
Lipid accumulation reduction activities of the candidate plant extracts. *Brucea javanica* (positive control) was used at 0.05 mg/mL. Data are expressed as means ± standard deviation. Means with different letters are significantly different (Tukey’s test, *p* < 0.05).

The abilities of the candidate plant extracts to reduce lipid accumulation were evaluated at concentrations of 0.05, 0.1, and 0.2 mg/mL (Figure 4). The candidate plants were divided into two groups based on their potential to reduce lipid accumulation in adipocytes (yes or no). *M. alba*, *C. nardus*, and *C. rotundus* exhibited no ability to reduce lipid accumulation, whereas the remaining plants displayed the potential to reduce lipid accumulation. Among them, *C. sappan* exhibited constant activity at all tested concentrations, whereas the effects of *A. concinna*, *E. longifolia*, and *T. triandra* increased with the increasing concentration.

#### 2.2.4. Cell Viability

**Figure 5:**
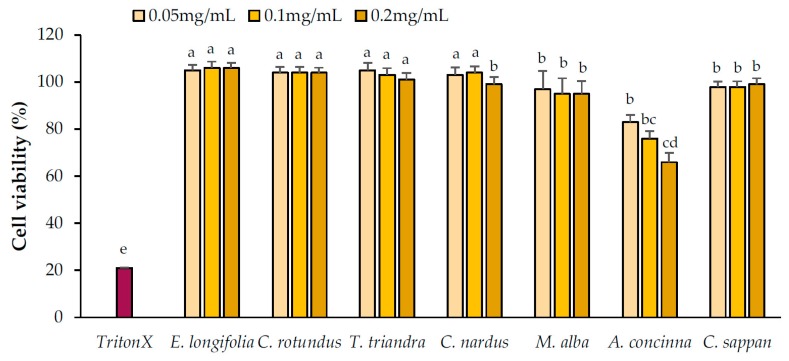
Cell viability after treatment with the candidate plant extracts. Triton X-100 (positive control) was used at 0.1% *v*/*v*. Data are expressed as means ± standard deviation. Means with different letters are significantly different (Tukey’s test, *p* < 0.05).

To observe the toxicity of medicinal plant extracts in adipocytes, cell viability after treatment with the plant extracts was examined (Figure 5). Excluding weak toxicity for *A. concinna*, none of the extracts were toxic at the tested concentrations.

## 3. Discussion

Numerous studies have focused on screening medicinal plants for effects against various diseases. In general, a single bio-assay is employed for this screening. The present study employed multiple assays to investigate both the digestion of lipids in the gastrointestinal tract and lipid synthesis/metabolism in adipocytes, both of which are important processes in the development of obesity (Table 2).

Among 70 Thai medicinal plants, seven medicinal plants namely, *M. alba*, *C. nardus*, *C. rotundus*, *C. sappan*, *E. longifolia*, *T. triandra*, and *A. concinna* were selected as candidates after the first round of screening. These medicinal plants were then subjected to a second round of screening using the same assays but at different concentrations, in addition to a cell viability assay, to select promising candidates.

Despite being selected as a candidate in the first round of screening, *M. alba* leaf extract displayed only moderate inhibitory effects on pancreatic lipase during the second round of screening, and the plant was not considered a preferred candidate in this study. However, this plant has been studied for its beneficial effects on obesity-related fatty liver disease [7]. In this prior study, *M. alba* extract was administered to high-fat diet (HFD)-fed mice for 12 weeks. Although there was no effect on body weight, the extract regulated hepatic lipid metabolism by attenuating liver X receptor α-mediated lipogenesis and upregulating lipolysis-associated markers such as lipoprotein lipase.

We found that *C. nardus* leaves exhibited moderate inhibitory effects on pancreatic lipase and strong lipolysis-enhancing effects. *C. nardus* leaves have been traditionally used in health products including soaps, anti-mosquito oils, candles, and pain-relieving oils [8]. Interestingly, a study by Batubara et al. reported the weight-reducing effects of citronella oil an essential oil extracted from *C. nardus* which contains *R*-citronellal, neryl acetate, citronellyl acetate, geraniol, and β-citronellol, in obese rats [8]. Inhalation of this essential oil led to increased sympathetic nerve activity and decreased appetite, weight gain, and cholesterol levels. The anti-obesogenic effects of consumption of this leaf have not yet been studied.

Next, *C. rotundus* rhizomes have been traditionally used to treat diabetes, pyrosis, inflammation, malaria, and stomach/gastrointestinal disorders [9]. The plant has not been used to treat obesity, but previous studies have investigated the effects of *C. rotundus* tubers on obesity using in vitro and in vivo methods [10,11]. Lamaure et al. administrated 45 or 220 mg/kg/day *C. rotundus* tuber hexane extract to Zucker rats for 60 days and observed a reduction in weight gain without an effect on food consumption [10]. In the same study, they described the ability of the extract to enhance lipolysis by 3T3-F442 adipocytes. In accordance with their result, *C. rotundus* rhizomes strongly enhanced lipolysis and weakly inhibited pancreatic lipase in the present study. Although reduced lipid accumulation was not observed in 3T3-L1 adipocytes in our experiment, the combination of lipase inhibition and lipolysis enhancement might be important for the reduction of weight gain observed in the study by Lamaure et al.

Furthermore, *C. sappan* heartwood strongly inhibited pancreatic lipase activity and reduced lipid accumulation reduction at all tested concentrations in the present study. Due to the lack of effects on lipolysis, the inhibitory effects of the extract on lipid accumulation are assumed to arise from the reduction of lipid synthesis in 3T3-L1 cells. The constituents in this plant have been previously studied. The heartwood of *C. sappan* contains flavonoids such as protosappanin and hematoxylin, and the main constituent among them is brazilin [12]. Brazilein, the dehydrogenated form of brazilin, was also isolated from the heartwood of *C. sappan* and was demonstrated to have anti-obesogenic activity. In particular, the compound inhibited the differentiation of 3T3-L1 preadipocytes through suppressing the induction of peroxisome proliferator-activated receptor γ, the master regulator of adipogenesis [13]. Together with our current result illustrating its potential to regulate lipid digestion and synthesis, *C. sappan* is considered a promising candidate for the treatment of obesity.

Moreover, *E. longifolia* roots displayed low pancreatic lipase-inhibitory activity but strong potential to enhance lipolysis and reduce lipid accumulation in 3T3-L1 adipocytes. *E. longifolia* is a well-known medicinal plant in the Asian region, and several studies have described the potential of its roots to treat obesity [14,15,16]. For instance, our research group previously explored the bioactive compounds of *E. longifolia* roots and their lipolysis-enhancing activities in a cell-based assay [15]. In the report, we identified eurycomanone and 13β,21-epoxyeurycomanone as the bioactive compounds in the roots of *E. longifolia*. The two quassinoids enhanced lipolysis with EC_50_s of 14.6 and 8.6 μM, respectively. An in vivo study also demonstrated the efficacy of this plant. The study found that a standardized quassinoid-enriched fraction of *E. longifolia* reduced body weight gain, adipose tissue hypertrophy, and serum glycerol levels in mice with HFD induced obesity [16]. Furthermore, clinical testing illustrated that consumption of the *E. longifolia* extract together with exercise enhanced lipolysis [14]. Thus, in addition to being identified as a promising candidate in this study, *E. longifolia* has been proven effective for the treatment of obesity by several researchers.

The last two candidates, *T. triandra* roots and *A. concinna* pods, are interesting candidates for anti-obesity purposes given that they have not been studied for this indication previously. The roots of *T. triandra* are traditionally employed for the treatment of cancer, fever, and malaria, but not obesity [17]. Conversely, the leaf extract of *T. triandra* has anti-obesogenic potential based on its ability to inhibit pancreatic lipase activity with an IC_50_ of 273.5 µg/mL [18]. Thus, our findings regarding the potential of the roots of the plant to inhibit pancreatic lipase, stimulate lipolysis, and reduce lipid accumulation in adipocytes should increase its potential utility for the treatment and prevention of obesity. There has been no report on the anti-obesogenic effects of *A. concinna*. Our result that *A. concinna* pods strongly inhibit pancreatic lipase, weakly stimulate lipolysis, and reduce lipid accumulation in adipocytes is a novel finding for this plant. Although *A. concinna* was found to be cytotoxic at high concentrations, it can be a good candidate for treating obesity at limited doses.

In conclusion, we screened 70 Thai medicinal plants using three in vitro methods for their anti-obesogenic potential, and seven plants were selected as candidates. Each candidate is expected to emerge as a potential candidate for obesity treatment. In particular, *T. triandra*, *E. longifolia*, and *A. concinna* exhibited activity in all three assays, suggesting their greater potential. In fact, the potential of *E. longifolia* has been demonstrated in in vivo experiments. Through further study of the other two plants, we hope to reveal that the combined in vitro assay is effective for screening potential medicinal plants and clarify the potential of *T. triandra* and *A. concinna* for treating obesity.

## 4. Materials and Methods

### 4.1. Preparation of Medicinal Plant Extracts

Seventy dried Thai medicinal plants were purchased from a local store in Samut Prakan, Thailand. The plant samples were stored at the Ministry of Public Health, Department of Thai Traditional and Alternative Medicine (Thailand), and voucher specimens (authentic crude drug) were issued for use as the medicinal plants’ species references. The plants were ground into fine small particles, and 5 g of each sample were soaked in 50% (*v*/*v*) aqueous methanol for 3 days. The supernatant of each suspension was filtered through filter paper, and the filtrates were evaporated to obtain the extracts used in the assays.

### 4.2. Pancreatic Lipase Inhibition Assay

Lipase inhibition was examined using Han’s method with modifications [19]. An emulsified solution of l-α-lecithin (Sigma-Aldrich, St. Louis, MO, USA; P4279, 10 mg), triolein (16 mg), and sodium taurocholate (5 mg) in Tris buffer (13 mM Tris, 150 mM NaCl, 1.3 mM CaCl_2_, pH 8.0, 9.0 mL) was used as a substrate. Porcine pancreatic lipase (Sigma, L3126, 4.5 mg) was dissolved in Tris buffer (30 mL) and used as an enzyme solution. The substrate (200 μL) and sample (100 μL, dissolved in 50% (*v*/*v*) aqueous dimethyl sulfoxide (DMSO)) were preincubated at 37 °C for 5 min. The reaction was started by adding the enzyme solution (100 μL) and allowed to continue for 30 min at 37 °C. The reaction was then stopped by adding 1 M hydrochloric acid (20 μL), and the liberated oleic acids were extracted using by hexane (600 μL). The hexane layer (300 μL) was dried and dissolved in DMSO (100 μL), and the oleic acid content was quantitated using a NEFA C-test Wako kit (Fujifilm Wako Pure Chem. Ind. Ltd., Osaka, Japan). Cetilistat (8 μM) was used as a positive control. The experiments were conducted in duplicate and repeated at least twice, and the representative values (mean ± standard deviation (SD)) are presented in the figures and tables.

### 4.3. Culture and Differentiation of 3T3-L1 Cells

Murine 3T3-L1 cells (JCRB9014) were obtained from the Japanese Collection of Research Bioresources Cell Bank (Osaka, Japan). 3T3-L1 cells were cultured in 96-well plates to confluence using basic medium (Dulbecco’s modified Eagle’s medium (DMEM) containing 10% (*v*/*v*) fetal bovine serum, 100 U/mL penicillin G potassium salt, 100 μg/mL streptomycin sulfate, and 50 μg/mL gentamicin sulfate). The cells were grown at 37 °C in a 10% carbon dioxide atmosphere. One day after reaching confluence, which was designated Day 0, cells were induced to undergo differentiation by changing the medium to differentiation medium, which was the basic medium containing 0.5 mM 3-isobutyl-1-methylxanthine (IBMX), 0.25 µM dexamethasone, and 10 µg/mL insulin. The medium was changed to insulin medium (the basic medium supplemented with 0.5 μg/mL insulin) on Days 2–4. From Day 4, the cell treatment differed between the lipolysis enhancement and lipid accumulation reduction assays.

### 4.4. Lipolysis Enhancement Assay

Cells were maintained in basic medium from Day 6 until Day 8. The plant extracts (dissolved in 10% (*v*/*v*) DMSO) were diluted with DMEM without phenol red and added to the cells. After 24 h of incubation, the lipolysis-enhancing activity of each extract was measured by quantitating the content of glycerol released from adipocytes into the medium. The supernatants were taken, mixed with free glycerol reagent (Sigma-Aldrich), and subjected to measurement according to the manufacturer’s instructions. The absorbance was measured at 540 nm using a Synergy™ MX microplate reader (BioTek Instruments, Inc., Winooski, VT, USA). Isoproterenol hydrochloride and 0.1% DMSO were used as the positive and negative controls, respectively. The experiments were repeated at least twice (*n* = 8 each), and the representative values (mean ± SD) are presented in the figures and tables. Lipolysis enhancement activity (% increase relative to control) was calculated according to the following formula:

(1) $$\%\;increase\;to\;control=\left\{\left(\frac{A_{540}Sample-A_{540}Blank}{A_{540}Control-A_{540}Blank}\right)-1\right\}\times 100.$$

### 4.5. Lipid Accumulation Suppression Assay

The plant extracts (dissolved in 10% (*v*/*v*) DMSO) were diluted in insulin-containing medium and added to the cells on Day 4. Then, the plant extracts were diluted in basic medium and added to the cells again on Day 6, followed by 3 days of incubation. The lipid content of the adipocytes was measured using AdipoRed™ reagent (LONZA, Tokyo, Japan). *Brucea javanica* extract (0.05 mg/mL), which is known to reduce lipid accumulation, was used as the positive control [20], and 0.1% DMSO was used as the negative control. The experiments were repeated at least twice (*n* = 8 each), and representative values (mean ± SD) are presented in the figures and tables. Lipid accumulation reduction activity (% decrease relative to control) was calculated as follows:

(2) $$\%\;decrease\;of\;control=\left\{1-\left(\frac{A_{540}Sample}{A_{540}Control}\right)\right\}\times 100.$$

### 4.6. Cell Viability Assay

Cell viability was measured using Cell Counting Kit-8 (CCK-8; Dojindo Lab., Kumamoto, Japan) in accordance with the manufacturer’s instructions. In brief, following 24 h of treatment with each extract, cells were incubated with CCK-8 reagent for 3 h. The absorbance of the supernatant was measured at 540 nm. Triton X-100 was used as the positive control for reference for cytotoxicity measurements. The experiments were repeated at least twice (*n* = 8 each), and representative values are presented in the figures or tables.

### 4.7. Statistics

The obtained data were statistically analyzed by one-way analysis of variance coupled with Tukey’s test. Differences were considered statistically significant at *p* < 0.05.

## Figures and Tables

**Table 1:** List of the selected Thai medicinal plants.

Scientific Name [6]	Voucher Specimens (Authentic Crude Drug)		Local Name	Family	Part Used
	Collector Number	TTM-c ^1^ Number			
*Acacia concinna* (Willd.) DC.	WR063	10000621	Som poi	Fabaceae	Pods
*Allium sativum* L.	WR001	10000559	Kra thiam	Amaryllidaceae	Clove
*Amomum testaceum* Ridl.	WR014	10000572	Krawan	Zingiberaceae	Seeds
*Andrographis paniculata* (Burm.f.) Nees	WR025	10000583	A thalai chon	Acanthaceae	Branches and leaves
*Angelica sinensis* (Oliv.) Diels	WR049	10000607	Kot chiang	Apiaceae	Roots
*Averrhoa carambola* L.	WR070	10000628	Ma fueang	Oxalidaceae	Branches and bark
*Azadirachta indica* A.Juss.	WR062	10000620	Sadao	Meliaceae	Branches
*Baliospermum solanifolium* (Burm.) Suresh	WR066	10000624	Tong tag	Euphorbiaceae	Roots
*Barleria lupulina* Lindl.	WR027	10000585	Salet phang phon	Acanthaceae	Branches and leaves
*Boesenbergia rotunda* (L.) Mansf.	WR043	10000601	Krachai	Zingiberaceae	Roots
*Bridelia ovata* Decne.	WR048	10000606	Ma ka	Phyllanthaceae	Branches and leaves
*Butea superba* Roxb.	WR040	10000598	Kwao khruea	Fabaceae	Bark
*Caesalpinia sappan* L.	WR067	10000625	Fang	Fabaceae	Heartwood
*Carthamus tinctorius* L.	WR004	10000562	Kham foi	Asteraceae	Flower
*Centella asiatica* (L.) Urb.	WR022	10000580	Bua bok	Apiaceae	Stems
*Chromolaena odorata* (L.) R.M.King and H.Rob.	WR023	10000581	Sap suea	Asteraceae	Branches
*Cinnamomum iners* Reinw. ex Blume	WR053	10000611	Op choei thai	Lauraceae	Branches
*Cissus quadrangularis* L.	WR020	10000578	Phet sang khat	Vitaceae	Bark
*Citrus aurantiifolia* (Christm.) Swingle	WR058	10000616	Ma nao	Rutaceae	Leaves
*Citrus hystrix* DC.	WR052	10000610	Ma krut	Rutaceae	Skin
*Cladogynos orientalis* Zipp. ex Span.	WR051	10000609	Chetta phang khi	Euphorbiaceae	Roots
*Cleome viscosa* L.	WR050	10000608	Phak sian phi	Cleomaceae	Branches and leaves
*Clitoria ternatea* L.	WR009	10000567	Anchan	Fabaceae	Flowers
*Croton fluviatilis* Esser	WR068	10000626	Plao noi	Euphorbiaceae	Trunk and bark
*Croton persimilis* Müll.Arg.	WR069	10000627	Plao yai	Euphorbiaceae	Trunk and bark
*Curcuma aeruginosa* Roxb.	WR033	10000591	Wan maha mek	Zingiberaceae	Rhizomes
*Cymbopogon citratus* (DC.) Stapf	WR061	10000619	Ta khrai	Poaceae	Stems
*Cymbopogon nardus* (L.) Rendle	WR037	10000595	Ta khrai hom	Poaceae	Leaves
*Cyperus rotundus* L.	WR056	10000614	Haeo mu	Cyperaceae	Rhizomes
*Derris elliptica* (Wall.) Benth.	WR065	10000623	Hang lai daeng	Fabaceae	Bark
*Dracaena cochinchinensis* (Lour.) S.C.Chen	WR054	10000612	Chan daeng	Asparagaceae	Bark
*Erythrina subumbrans* (Hassk.) Merr.	WR044	10000602	Thong lang	Fabaceae	Bark
*Eurycoma longifolia* Jack	WR041	10000599	Pla lai phueak	Simaroubaceae	Roots
*Glycyrrhiza glabra* L.	WR046	10000604	Cha em	Fabaceae	Branches
*Helicteres isora* L.	WR003	10000561	Po bit	Malvaceae	Pods
*Hibiscus sabdariffa* L.	WR002	10000560	Krachiap daeng	Malvaceae	Flower
*Hydnophytum formicarum* Jack	WR059	10000617	Hua roi ru	Rubiaceae	Trunk
*Jasminum sambac* (L.) Aiton	WR057	10000615	Mali	Oleaceae	Flowers
*Lagerstroemia speciosa* (L.) Pers.	WR021	10000579	Inthanin	Lythraceae	Leaves
*Mimosa pudica* L.	WR007	10000565	Maiyarap	Fabaceae	Branches and leaves
*Momordica charantia* L.	WR012	10000570	Ma ra khi nok	Cucurbitaceae	Branches and leaves
*Morinda citrifolia* L.	WR060	10000618	Yo	Rubiaceae	Fruit
*Moringa oleifera* Lam.	WR013	10000571	Marum	Moringaceae	Seeds
*Morus alba* L.	WR034	10000592	Mon	Moraceae	Leaves
*Nelumbo nucifera* Gaertn.	WR045	10000603	Bua luang	Nelumbonaceae	Stamens
*Ocimum tenuiflorum* L.	WR035	10000593	Ka phrao	Lamiaceae	Branches
*Orthosiphon aristatus* (Blume) Miq.	WR006	10000564	Ya nuat maeo	Lamiaceae	Branches and leaves
*Oxyceros horridus* Lour.	WR029	10000587	Khat khao	Rubiaceae	Fruits
*Paederia linearis* Hook.f.	WR032	10000590	Tot mu tot ma	Rubiaceae	Branches
*Peltophorum pterocarpum* (DC.) K.Heyne	WR047	10000605	Khi lek	Fabaceae	Core
*Phyllanthus emblica* L.	WR016	10000574	Ma kham pom	Phyllanthaceae	Seeds
*Physalis angulata* L.	WR024	10000582	Thong theng	Solanaceae	Barks
*Piper nigrum* L.	WR030	10000588	Phrik thai	Piperaceae	Seeds
*Piper retrofractum* Vahl	WR031	10000589	Di pli	Piperaceae	Fruits
*Piper sarmentosum* Roxb.	WR010	10000568	Cha phlu	Piperaceae	Branches and leaves
*Pluchea indica* (L.) Less.	WR026	10000584	Khlu	Asteraceae	Branches and leaves
*Pueraria candollei* var. *mirifica* (Airy Shaw and Suvat.) Niyomdham	WR039	10000597	Kwao khruea khao	Fabaceae	Bark
*Rauvolfia serpentina* (L.) Benth. ex Kurz	WR019	10000577	Rayom	Apocynaceae	Roots
*Rhinacanthus nasutus* (L.) Kurz	WR008	10000566	Thong pharchang	Acanthaceae	Branches and leaves
*Solanum sanitwongsei* W. G. Craib	WR015	10000573	Ma waeng	Solanaceae	Branches and leaves
*Syzygium aromaticum* (L.) Merr. and L.M.Perry	WR028	10000586	Kan phlu	Myrtaceae	Buds
*Tectona grandis* L.f.	WR018	10000576	Sak	Lamiaceae	Bark
*Terminalia chebula* Retz.	WR017	10000575	Samo thai	Combretaceae	Seeds
*Thunbergia laurifolia* Lindl.	WR011	10000569	Rang chuet	Acanthaceae	Bark
*Tiliacora triandra* Diels	WR036	10000594	Ya nang	Menispermaceae	Roots
*Tinospora crispa* (L.) Hook. f. and Thomson	WR005	10000563	Bora phet	Menispermaceae	Branches and leaves
*Ventilago denticulata* Willd.	WR038	10000596	Rang daeng	Rhamnaceae	Bark
*Ventilago denticulata* Willd.	WR064	10000622	Rang daeng	Rhamnaceae	Core
*Wrightia arborea* (Dennst.) Mabb.	WR055	10000613	Mok man	Apocynaceae	Bark
*Zingiber montanum* (J.Koenig) Link ex A.Dietr.	WR042	10000600	Phlai	Zingiberaceae	Rhizomes

^1^ Thai traditional medicine crude drug.

**Table 2:** Summary of the anti-obesogenic potential of the candidate plants.

Medicinal Plants	Pancreatic Lipase Inhibition	Lipolysis Enhancement	Lipid Accumulation Reduction	Toxicity
*M. alba*	Medium	NA *	NA	Negative
*T. triandra*	Low	Weak	Active (CD *)	Negative
*C. nardus*	Medium	Strong	NA	Negative
*E. longifolia*	Low	Strong	Active (CD)	Negative
*C. rotundus*	Low	Strong	NA	Negative
*A. concinna*	High	Weak	Active (CD)	Positive
*C. sappan*	High	NA	Active (constant)	Negative

* NA: no activity, CD: concentration dependent.

## Supplementary Materials

The following are available online at https://www.mdpi.com/1424-8247/13/4/56/s1, Table S1, Anti-obesogenic potential of Thai medicinal plants.
