# Supplementary material for: Selection of Thai Medicinal Plants with Anti-Obesogenic Potential via In Vitro Methods
Source: Pharmaceuticals (Basel). 2020 Mar 29;13(4):56. doi: 10.3390/ph13040056 (PMC7243097; doi:10.3390/ph13040056)
Supplement: Supplementary file 1 [file pharmaceuticals-13-00056-s001.pdf]

Table S1. Anti-obesogenic potential of Thai medicinal plants

Medicinal plants	Lipase inhibition (%) at 0.5 mg/mL	Lipolysis enhancement (% increase of control) at 0.5 mg/mL	Lipid accumulation reduction (% decrease to control) at 0.5 mg/mL
Acacia concinna (Willd.) DC.	100±0.1	48±5	1±1.5
Allium sativum L.	11±0.6	17±5	7±1.5
Amomum testaceum Ridl.	69±4.0	16±8	-1±4.5
Andrographis paniculata (Burm.f.) Nees	88±4.3	23±6	-2±1.2
Angelica sinensis (Oliv.) Diels	40±2.1	-49±6	-3±1.3
Averrhoa carambola L.	0±1.1	-17±11	6±
Azadirachta indica A.Juss.	-11±6.2	-8±6	1±1.3
Baliospermum solanifolium (Burm.) Suresh	11±5.7	17±6	1±0.8
Barleria lupulina Lindl.	55±5.6	-34±2	-12±1.7
Boesenbergia rotunda (L.) Mansf.	63±1.3	3±2	6±1.4
Bridelia ovata Decne.	91±0.1	-62±7	-2±1.1
Butea superba Roxb.	-25±6.6	-1±6	-4±1.0
Caesalpinia sappan L.	74±0.9	-17±2	12±1.6
Carthamus tinctorius L.	29±0.3	10±5	2±2.3
Centella asiatica (L.) Urb.	38±9.7	4±7	3±2.0
Chromolaena odorata (L.) R.M.King & H.Rob.	17±0.7	35±12	6±2.3
Cinnamomum iners Reinw. ex Blume	47±2.6	24±10	-3±1.4
Cissus quadrangularis L.	15±1.2	34±11	9±3.7
Citrus aurantiifolia (Christm.) Swingle	6±0.8	0±5	4±1.1
Citrus hystrix DC.	41±1.8	10±3	1±1.3
Cladogynos orientalis Zipp. ex Span.	79±7.7	10±2	-2±1.1
Cleome viscosa L.	62±3.9	4±13	-1±1.4
Clitoria ternatea L.	16±3.0	20±6	1±1.4
Croton fluviatilis Esser	39±3.5	-6±2	-2±1.1
Croton persimilis Müll.Arg.	35±9.6	-15±1	-1±1.5
Curcuma aeruginosa Roxb.	91±0.3	-26±5	-2±3.1

Cymbopogon citratus (DC.) Stapf	20±1.6	3±4	3±1.5
Cymbopogon nardus (L.) Rendle	85±3.5	55±5	-18±1.3
Cyperus rotundus L.	68±4.2	39±10	15±3.0
Derris elliptica (Wall.) Benth.	-26±7.9	5±6	5±0.7
Dracaena cochinchinensis (Lour.) S.C.Chen	72±0.1	-32±11	1±0.9
Erythrina subumbrans (Hassk.) Merr.	62±3.5	-5±5	3±2.3
Eurycoma longifolia Jack	50±3.0	85±4	12±1.6
Glycyrrhiza glabra L.	79±2.1	25±8	6±2.4
Helicteres isora L.	31±1.3	1±5	8±1.3
Hibiscus sabdariffa L.	10±2.3	13±5	6±1.4
Hydnophytum formicarum Jack	48±1.2	-20±4	3±1.0
Jasminum sambac (L.) Aiton	-11±5.4	11±7	10±3.6
Lagerstroemia speciosa (L.) Pers.	77±5.7	-35±12	4±1.6
Mimosa pudica L.	15±3.8	19±6	1±1.9
Momordica charantia L.	71±3.5	21±8	-3±2.0
Morinda citrifolia L.	16±1.3	0±6	0±1.4
Moringa oleifera Lam.	47±0.6	2±7	2±2.5
Morus alba L.	87±2.4	-21±2	10±2.1
Nelumbo nucifera Gaertn.	26±0.4	14±2	1±2.7
Ocimum tenuiflorum L.	45±2.4	-2±8	11±1.6
Orthosiphon aristatus (Blume) Miq.	22±3.5	-2±8	1±1.3
Oxyceros horridus Lour.	26±4.7	-28±6	-10±3.9
Paederia linearis Hook.f.	12±0.9	-24±2	5±2.0
Peltophorum pterocarpum (DC.) K.Heyne	66±1.3	-28±9	3±1.2
Phyllanthus emblica L.	15±9.0	-3±13	3±2.7
Physalis angulata L.	41±6.6	21±13	-3±2.3
Piper nigrum L.	14±5.4	-22±6	0±2.6
Piper retrofractum Vahl	62±0.6	-30±3	3±2.5
Piper sarmentosum Roxb.	11±5.3	21±7	6±2.6
Pluchea indica (L.) Less.	74±1.1	-48±6	0±1.8

Pueraria candollei var. *mirifica* (Airy Shaw & Suvat.) Niyomdham	-6±1.8	-13±5	-8±0.7
Rauvolfia serpentina (L.) Benth. ex Kurz	1±5.4	32±9	1±2.7
Rhinacanthus nasutus (L.) Kurz	6±2.8	9±5	1±1.4
Solanum sanitwongsei W. G. Craib	21±2.7	24±9	-3±1.9
Syzygium aromaticum (L.) Merr. & L.M.Perry	67±0.8	-34±5	-8±1.7
Tectona grandis L.f.	6±15.0	14±8	5±2.6
Terminalia chebula Retz.	13±0.3	30±10	5±2.3
Thunbergia laurifolia Lindl.	55±0.3	27±9	8±1.9
Tiliacora triandra Diels	49±3.3	74±2	14±2.8
Tinospora crispa (L.) Hook. f. & Thomson	61±1.8	15±6	2±1.0
Ventilago denticulata Willd.	96±1.6	-15±5	-11±1.2
Ventilago denticulata Willd.	29±4.2	1±5	2±1.5
Wrightia arborea (Dennst.) Mabb.	74±0.5	12±5	2±2.5
Zingiber montanum (J.Koenig) Link ex A.Dietr.	84±1.3	6±5	0±1.1
